# The Relationship between Environmental Awareness, Habitat Quality, and Community Residents’ Pro-Environmental Behavior—Mediated Effects Model Analysis Based on Social Capital

**DOI:** 10.3390/ijerph192013253

**Published:** 2022-10-14

**Authors:** Wentao Si, Chen Jiang, Lin Meng

**Affiliations:** School of Public Administration, Shandong Normal University, Jinan 250014, China

**Keywords:** environmental awareness, habitat quality, social capital, pro-environmental behavior, community residents, Jinan, China

## Abstract

Pro-environmental behavior can promote the optimization of the living environment and sustainable social development. This paper constructs a theoretical analysis framework of “environmental consciousness, habitat environment-social capital- pro-environmental behavior”. By using structural equation modeling and 1005 instances of microscopic research data, we analyzed the influence of environmental awareness and habitat environment on the pro-environmental behavior of community residents. The results of the analysis were combined with the Bootstrap method to verify the mediating role of social capital dimensions in the influence of environmental awareness and habitat quality on the pro-environmental behavior of community residents. The results show that: Firstly, environmental awareness, habitat quality, and social capital have positive effects on the pro-environmental behavior of community residents. Secondly, environmental awareness and habitat quality have positive effects on the five dimensions of social capital. Thirdly, among the five dimensions of social capital, four dimensions of social trust, social norms, sense of community belonging, and community voluntarism play a partially mediating role between environmental awareness, habitat quality, and pro-environmental behavior. This paper enriches the research on the influence of environmental awareness and habitat environment on pro-environmental behavior, reveals the mediating effect of each dimension of social capital, and broadens the horizon for the study of pro-environmental behavior. The results of the study can provide a reference for decision making to promote the implementation of pro-environmental behavior among community residents.

## 1. Introduction

Pro-environmental behavior, also known as environmentally friendly behavior or environmental protection behavior, is a socially altruistic behavior of community residents who have a positive attitude and behavioral tendency to care about the environment [1]. The concept of pro-environmental behavior is based on the relationship of “Individuals have an impact on the environment”. The elements that influence environmentally friendly behavior can be divided into four categories.

The first type of influencing factors are cognitive factors, namely people’s environmental awareness [2], environmental knowledge [3], and so on. At the same time, it also includes the personality traits of individuals. The agreeableness and openness in personality traits are considered to be the two personality traits most related to the pro environmental behavior [4].

The second influencing factor is the emotional factor, which can be divided into two parts in an irrational perspective. On the one hand, this kind of research focused on the influence of individual emotions on the willingness to practice pro environmental behavior [5]. On the other hand, it focused on the influence of the emotional connection between individuals and specific things on the implementation of pro environmental behavior. Some scholars have found that human feelings about the current place of residence will affect individuals’ behavior in public life. At the same time, human beings connect with the environment through the place where they live, and then have a place attachment to the place where they live [6].

The third kind of influencing factor is social psychological factor, which includes ethics, environmental attitude, and environmental values. Moral norm refers to the moral cognition that individuals embody when they make certain behavioral decisions [7]. The social pressures they bring have an impact on individuals’ pro-environment behavior [8]. Environmental attitude refers to an individual’s subjective evaluation of a behavior, which presents both positive and negative aspects. Numerous empirical studies have shown that environmental behavior attitudes have a significant positive effect on individuals practicing pro-environmental behavior [1]. Environmental values are the guiding principles that individuals have developed, and this guiding principle, as an intrinsic element, profoundly influences pro-environmental behavior [9].

The fourth influencing factor is the situational factor. Excluding cognitive, emotional, psychosocial, and other elements, the academic community also pointed out that situational factors have an important impact on pro-environmental behavior. The availability of convenient transportation facilities [10], the availability of facilities to recycle waste [11], the availability of energy-efficient commodities for sale, and the availability of suitable prices [12] all affect an individual’s willingness and motivation to engage in pro-environmental behavior.

In summary, residents’ pro-environmental behavior is a behavior driven by the joint action of their own emotional factors and the external environment [13], which is the performance of both internal and external factors. The mechanism of the interaction between internal motivational factors and external influences, which is the key factor in establishing a long-term mechanism for environmental protection, has been little explored by scholars. This study aims to investigate the multiple effects of environmental awareness as an internal personal psychological factor, habitat quality as an external contextual factor, and social capital as a social environmental factor on pro-environmental behavior.

From the perspective of residents’ own emotional factors, their internal recognition and support of pro-environmental awareness as independent-minded individuals is the psychological basis for the implementation of pro-environmental behaviors [14]. Environmental consciousness can be applied to residents’ own behavior through their evaluation and judgment of external objective things, which is also a necessary condition to ensure the long-term implementation of pro-environmental behavior [14]. From the perspective of external factors, the community residents’ behavior will be affected by the external situational factors, namely habitat environment [15]. The evaluation of the quality of the habitat environment is the subjective feelings and psychological perceptions of residents regarding the community environment and the changes in this environment, which can influence people’s specific environmental behaviors in a certain contextual environment [16]. At the same time, social capital as one of the social environmental factors can also have some influence on residents’ pro-environmental behaviors [17]. For example, when individual cognition and moral beliefs are consistent with group norms, individuals will identify with the group and be influenced by it. If the group is related to the protection of the environment, its members are more likely to engage in pro-environmental behavior [18]. Thus, social capital in the social environment may be a factor that attracts members to participate and act, and can positively influence pro-environmental behavior.

### 1.1. Theoretical Assumptions and Analysis

#### 1.1.1. The Direct Influence of Environmental Awareness on Pro-Environmental Behavior

According to “Social Cognitive Theory”, the vast majority of individual behaviors are goal-oriented. This means that when individuals have a higher level of cognition, the quality of personal behavior will be correspondingly higher, and vice versa [19]. Similarly, environmental awareness has a very important role in influencing the pro-environmental behavior of community residents. Individuals with higher levels of environmental concern are more inclined toward pro-environmental behavior [20,21]. That is to say, if residents have a higher level of environmental concern, their willingness to engage in pro-environmental behavior will be stronger [22]. In addition, environmental concern also has a positive effect on individuals’ willingness to pay green [23]. Furthermore, the more environmentally knowledgeable residents are, the more knowledgeable and skilled they are to implement pro-environmental behaviors, thus promoting them to implement pro-environmental behaviors consciously will be easier [24]. Finally, establishing correct environmental values not only can effectively reduce the negative impact of their own behavior on the environment [25], but also can motivate them to take corresponding measures to implement environmental protection and improve their behavior. On the contrary, low environmental awareness inhibits residents’ knowledge about the environment to a certain extent, restricts their participation enthusiasm, and hinders the implementation of pro-environmental behaviors in general [26].

#### 1.1.2. Direct Impact of Habitat Quality on Pro-Environmental Behavior

Contextual factors such as the quality of human habitat also have an impact on pro-environmental behavior [27]. The affluence theory of economic development suggests that the affluence of a region is positively related to environmental responsibility and the pro-environmental behavior of local residents. The environment is not only a public good, but also a “scarce good”. Therefore, as economic growth increases the demand for environmental improvement, people will consume the “scarce goods” after necessary consumption [28]. “Low-cost Theory “suggests that people are more likely to behave in an environmentally manner in situations where they have lower costs of action. The term “cost” here refers to a broad sense of “cost”, i.e., not only economic costs, but also non-economic costs, such as the convenience of the living environment and difficulty of implementation.

Based on the above theoretical analysis and research, it can be determined that the quality of the habitat environment can have an impact on pro-environmental behavior in the following aspects. First, the quality of environmental hygiene can have an impact on residents’ pro-environmental behavior. Specifically, in the process of living, residents will regard the environmental health condition of the community as an external environmental signal, and based on the interpretation of this signal, they will formulate community behavior rules, i.e., whether or not the behavior is acceptable, and make their self-behavior choices accordingly [21]. Likewise, the convenience, comfort, and safety of the infrastructure can influence the willingness and motivation of residents to participate in environmental actions. A clear example is Switzerland, where a virtuous cycle of waste recycling has been developed, with clear responsibilities and division of labor, both for sorting, collection, and reuse. The reason for the high level of waste recycling behavior among Swiss community residents is that these areas already provide convenient waste recycling facilities, and all the residents have to do is to choose the right bins to put their waste in, a practice that increases the convenience of pro-environmental behavior [29]. Similarly, community environmental management has a greater impact on pro-environmental behavior. There will be more positive pro-environmental behavior in residents when the quality of residential environment management is higher and where there is a certain quality of life guarantee for the residents of the able residential area [30].

### 1.2. The Intermediary Role of Social Capital

#### 1.2.1. The Mediating Role of Social Capital between Environmental Awareness and Pro-Environmental Behavior

Residents’ environmental awareness has an influential role in the formation of social capital. Residents’ awareness of environmental issues affects their social participation, which in turn strengthens understanding, familiarity, and identification among community members, and creates a dense network of social relationships through continuous interaction [31]. Through communication, coordination, and interaction, residents in a social network can determine how to cooperate with each other in a mutually beneficial way [32]. The process of mutual agreement on goals and values lays the foundation for the cultivation of social trust [33], the core element of social capital. In the process of participation, the rules of “mutual aid” and “reciprocity” are formed [13], and a sense of community is established in real life and in a particular environment. As residents develop a sense of belonging and cohesion in the community, social capital can be formed in spaces where community members can interact.

At the same time, the formation of social capital can guide the collective action of community members, on this basis, become a factor to guide individual action in the community [34], and also promote the community to organize itself for voluntary services [32]. Pro-environmental behavior can be seen as an “Altruistic Behavior”, when people pay for the environment as a “Quasi-Public Good”, they tend to have a sense of “contribution”, because the positive externalities of environmental protection are often greater for the public domain than for the private domain [35]. This tends to create a sense of “empowerment”, and thus is more likely to promote the positive effects of social capital on environmental behavior [31].

#### 1.2.2. The Mediating Role of Social Capital between Habitat Quality and Pro-Environmental Behavior

The quality of habitat affects the formation of social capital. The quality of habitat indirectly reflects the economic status of the region and is the deep soil for cultivating the social capital of the residents [30]. If an area has a high level of economic development, then the improvement of environmental health, infrastructure, and environmental management can achieve a greater degree for community residents’ recognition of the quality of their living environment. In order to maintain the status quo, people consciously or unconsciously form organizations and reach a consensus, forming an atmosphere and force that can protect the status quo. In other words, when residents perceive a high-quality living environment, they will have positive feelings and influences on the development of social capital.

The development of social capital influences the implementation of pro-environmental behaviors. Community residents’ satisfaction with the quality of their habitat is implicitly internalized in their emotions toward the community. Emotions about the community lead residents to invest resources in their community and to engage in a range of beneficial behaviors for the development of the community [36]. It is also believed that residents are more willing to help others or to participate in community activities when they are satisfied with their living experience. Community residents are often able to form an environmental organization on environmental issues, and the development of environmental organizations is more mature, thus helping to guide people to become involved in environmental issues [37]. At the same time, good environmental management helps to resolve community frictions, thus enhancing the cohesiveness and solidarity of community residents.

Research proves that social capital can be an effective resource pool for collective community action through the long-term interaction of community residents or organizations [38]. At the same time, social capital as a “soft environment” can also improve the efficiency of social operation, become a catalyst for social vitality, and the habitat environment is the spatial carrier for the formation of social capital. The two are mutually coupled to promote the optimization of habitat. Under the influence of the social atmosphere, where residents advocate for environmental protection and effective interpersonal interaction, the habitat environment has more effective impacts on the pro-environmental behavior of individuals [30]. Social capital promotes residents’ participation in environmental governance, as well as promotes synergistic cooperation and benign interaction among environmental stakeholders based on trust in environmental governance, which plays a role in resolving people’s conflicts in environmental resource allocation and eases social conflicts [32]. Thus, social capital is considered as a more efficient and humane tool for organization and coordination in environmental governance. As mentioned earlier, habitat quality has positive implications for social capital, which in turn has an impact on pro-environmental behavior. Therefore, this study predicts that better habitat environment will lead to higher levels of social capital among residents, which in turn will promote their pro-environmental behavior.

### 1.3. Objectives and Hypotheses

**Figure 1:**
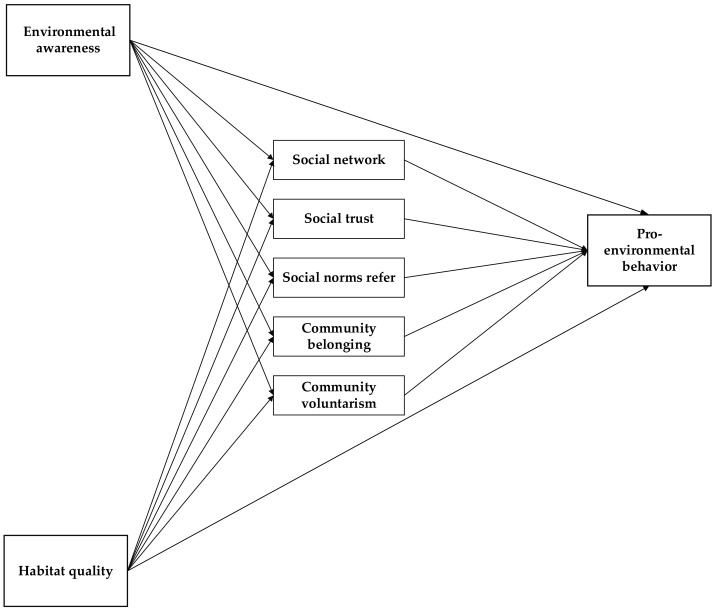
Theoretical framework diagram.

This study intends to construct a structural relationship model of “environmental awareness, habitat quality-social capital- pro-environmental behavior” (Figure 1) to investigate the relationship between environmental awareness, habitat quality, social capital, and pro-environmental behavior. In this study, we explore the mediating mechanism through which environmental awareness, habitat quality, and social capital influence the pro-environmental behaviors of community residents.

Based on previously reviewed research, the following hypotheses were proposed (Figure 1).

**Hypothesis 1 (H1).**  *Environmental awareness has a direct impact on the dimensions of social capital.*

**Hypothesis 1a (H.1a).**  *Environmental awareness has a direct impact on Social network.*

**Hypothesis 1b (H.1b).**  *Environmental awareness has a direct impact on Social trust plays.*

**Hypothesis 1c (H.1c).**  *Environmental awareness has a direct impact on Social norms refer.*

**Hypothesis 1d (H.1d).**  *Environmental awareness has a direct impact on Community belonging.*

**Hypothesis 1e (H.1e).**  *Environmental awareness has a direct impact on Community voluntarism.*

**Hypothesis 2 (H2).**  *Habitat quality has a direct impact on the dimensions of social capital.*

**Hypothesis 2a (H.2a).**  *Habitat quality has a direct impact on Social network.*

**Hypothesis 2b (H.2b).**  *Habitat quality has a direct impact on Social trust plays.*

**Hypothesis 2c (H.2c).**  *Habitat quality has a direct impact on Social norms refer.*

**Hypothesis 2d (H.2d).**  *Habitat quality has a direct impact on Community belonging.*

**Hypothesis 2e (H.2e).**  *Habitat quality has a direct impact on Community voluntarism*.

**Hypothesis 3 (H3).**  *Environmental awareness has a direct impact on pro-environmental behavior.*

**Hypothesis 4 (H4).**  *Habitat quality has a direct impact on pro-environmental behavior.*

**Hypothesis 5 (H5).**  *Social capital has a direct impact on pro-environmental behavior.*

**Hypothesis 5a (H.5a).**  *Social network has a direct impact on pro-environmental behavior.*

**Hypothesis 5b (H.5b).**  *Social trust has a direct impact on pro-environmental behavior.*

**Hypothesis 5c (H.5c).**  *Social norms refer has a direct impact on pro-environmental behavior.*

**Hypothesis 5d (H.5d).**  *Community belonging has a direct impact on pro-environmental behavior.*

**Hypothesis 5e (H.5e).**  *Community voluntarism has a direct impact on pro-environmental behavior.*

**Hypothesis 6 (H6).**  *Social capital has a mediating effect between the influence of environmental awareness and pro-environmental behavior.*

**Hypothesis 6a (H.6a).**  *Social network has a mediating effect between the influence of environmental awareness and pro-environmental behavior.*

**Hypothesis 6b (H.6b).**  *Social trust has a mediating effect between the influence of environmental awareness and pro-environmental behavior.*

**Hypothesis 6c (H.6c).**  *Social norms refer has a mediating effect between the influence of environmental awareness and pro-environmental behavior.*

**Hypothesis 6d (H.6d).**  *Community belonging has a mediating effect between the influence of environmental awareness and pro-environmental behavior.*

**Hypothesis 6e (H.6e).**  *Community voluntarism has a mediating effect between the influence of environmental awareness and pro-environmental behavior.*

**Hypothesis 7 (H7).**  *Social capital has a mediating effect between the effects of habitat quality and pro-environmental behavior.*

**Hypothesis 7a (H.7a).**  *Social network has a mediating effect between the effects of habitat quality and pro-environmental behavior.*

**Hypothesis 7b (H.7b).**  *Social trust has a mediating effect between the effects of habitat quality and pro-environmental behavior.*

**Hypothesis 7c (H.7c).**  *Social norms have a mediating effect between the effects of habitat quality and pro-environmental behavior.*

**Hypothesis 7d (H.7d).**  *Community belonging has a mediating effect between the effects of habitat quality and pro-environmental behavior*.

**Hypothesis 7e (H.7e).**  *Community voluntarism has a mediating effect between the effects of habitat quality and pro-environmental behavior.*

## 2. Data and Methodology

### 2.1. Methodology

This study applied AMOS 21.0 to examine and analyze the mechanisms underlying the influence of environmental awareness, habitat quality, and social capital on pro-environmental behavior. Each latent variable was measured via multiple entries in the scale. To further validate the mediating effects and influence mechanisms of social capital, the Bootstrap method was used to validate the mediating effects of each of the five dimensions of social capital.

### 2.2. Variable Selection

Based on the mature scales at home and abroad, the environmental awareness, habitat quality, social capital, and pro-environmental behavior scales were designed by combining the research objectives and by using the Likert 5-point scale.

#### 2.2.1. Independent Variable: Environmental Awareness

Environmental awareness refers to the intention of individuals to promote a series of environmental actions motivated by psychological factors and emotional forces [20]. In conjunction with the purpose of the study, the environmental awareness of community residents was divided into three dimensions: environmental concern, environmental knowledge, and environmental values [4]. Firstly, environmental concern is the degree of residents’ tendency to recognize environmental problems and to support the solutions to these problems [39]. Secondly, environmental knowledge refers to the specific knowledge mainly related to the protection of the environment, which is mainly reflected in the three aspects of system, action and utility [3]. Thirdly, environmental values are residents’ emotions, perceptions, and behavioral intentions toward environmental protection issues [9] (Table 1).

#### 2.2.2. Independent Variable: Habitat Quality

Habitat quality refers to the degree to which the environment is suitable for human living [40]. The comprehensive evaluation indexes of habitat quality can be divided into three aspects: environmental health conditions, infrastructure conditions, and environmental management conditions [41]. Firstly, environmental health conditions mainly includes the satisfactory outdoor air quality, water quality, and greening degree [42]. Secondly, infrastructure conditions refer to the municipal utilities within the community, including community greening conditions, environmental health conditions, municipal facilities deployment, and disaster prevention support facilities [41,42]. Thirdly, environmental management conditions include greening management, safety and disaster prevention management, and municipal facility management [41,42] (Table 2).

#### 2.2.3. Mediating Variable: Social Capital

Social capital is an important factor that effectively motivates individuals to actively participate in cooperative behavior [17]. The social capital discussed in this study refers to the social capital of community residents, which is analyzed in five dimensions: social network, social trust, social norms, sense of community belonging, and community voluntarism. Firstly, social network refers to a kind of “local social relationship network”, which is an individual social network that includes neighborhood relationships and interactions formed by community residents via community participation [43]. Secondly, social trust refers to a special trust relationship formed by the community residents in the process of long-term interaction [17]. Thirdly, social norms refer to the behavioral norms of community residents in maintaining generally acceptable community order, promoting the collective actions of community members, and in maximizing the collective welfare of community members [17]. Fourthly, community belonging refers to the emotional attachment of community residents to their communities [44]. Fifth, community voluntarism refers to the willingness of residents to volunteer in the community, and thus to help other residents without compensation for performing these helpful acts (Table 3).

#### 2.2.4. Dependent Variable: Pro-Environmental Behavior

Pro-environmental behavior is a conscious behavior that reflects the subject’s sense of personal and social responsibility, as well as environmental values in order to be able to solve environmental problems [45]. According to Sivek and Hungerford, the attributes of pro-environmental behavior determine it as a behavior that allows for the sustainable and controlled exploitation of natural resources [45]. According to Stern, pro-environmental behavior can be considered as an activity that aims to protect the ecological environment [41]. Hungerford and Sia et al., organized pro-environmental behavior into four dimensions: ecological management, consumption behavior, persuasive behavior, and citizenship behavior [42]. Among them, ecological management is a series of practical actions taken to maintain and protect the ecology or improve the environment; consumption behavior uses green consumption-oriented means to protect the environment; persuasive behavior means persuading or encouraging others to engage in positive environmental protection behavior through the subject’s words; civic behavior means taking the initiative to perform one’s civic duties and to pay attention to, and explore and discuss how to solve environmental issues (Table 4).

### 2.3. Study Area and Data Acquisition

Jinan is the capital city of Shandong Province and one of the 15 sub-provincial cities in China, with a per capita gross regional product of ¥110,119.00 in 2020 (the national per capita GDP was ¥71,999.60). The resident population of Jinan is 9,202,400, and the urbanization rate of the resident population reaches 77.00% (13.11% higher than the national average of 63.89%). Therefore, the study and discussion of Jinan city as a case area can be a reference value for exploring the pro-environmental behavior of residents in other large and medium-sized cities.

The empirical research data for this study were mainly obtained from the questionnaire. The study combines random sampling and stratified sampling to investigate the research. First, four districts in Jinan, namely, Lixia, Licheng, Changqing, and Zhangqiu, were selected according to the regional economic development status to conduct the survey. Among them, the economic development level of Lixia district is the highest in Jinan city, the economic development level of Licheng district and Zhangqiu district is in the middle, and the economic development level of Changqing district is relatively weak. Secondly, four residential areas were randomly selected in each district. Again, 50–80 residents were randomly selected in each sample residential area according to a certain proportion, and the questionnaire survey was conducted in a one-on-one question-and-answer format, and each questionnaire was guaranteed to be completed by one of the members of each household.

The data collection was divided into two stages: pre-survey and formal survey. A total of 150 questionnaires were collected in the pre-survey stage from July to September 2021, some of the items were improved after the reliability analysis, and the questionnaire questions were modified in a colloquial way to ensure the effectiveness of the questionnaire. A total of 1150 questionnaires were formally placed in the formal survey stage from September to December 2021, and 1005 questionnaires were effectively answered. The results of the descriptive statistics of the sample are shown in Table 5.

## 3. Empirical Tests and Results

### 3.1. Structural Equation Model Testing

#### 3.1.1. Reliability and Validity Tests

Four latent variables were set in this study’s scale, which was measured using 56 measurement questions. The overall Cronbach’s alpha for the environmental awareness variable was 0.846, and the Cronbach’s alpha for environmental concern, environmental knowledge, and environmental evaluation were 0.794, 0.831, and 0.807, respectively. The overall Cronbach’s alpha for the living environment variable was 0.884, the overall Cronbach’s alpha for the social capital variable was 0.887, and the Cronbach’s alpha for social network, social trust, social norms, community belonging, and community voluntarism were 0.869, 0.889, and 0.861, respectively. The overall Cronbach’s alpha for pro-environmental behavior variables was 0.826, and the Cronbach’s alpha for the four dimensions of pro-environmental behavior, ecological management, consumer behavior, persuasive behavior, and civic behavior, were 0.892, 0.796, 0.889, and 0.789, respectively. The coefficient values for each variable and each dimension were greater than 0.7; thus the reliability of the questionnaire in this study was good. In addition, the CITC values (corrected item total correlation) between the observed variables and their latent variables were greater than the standard value of 0.5, thus indicating that the survey options for each latent variable were well set, i.e., the questionnaire reliability was good.

#### 3.1.2. Structural Equation Model Fitting Index

According to the study hypothesis, with the help of AMOS 24.0 data analysis software, the X^2^/df value was 1.985, which is less than 3; the RMSEA was 0.031, which is less than the standard level of 0.08, indicating a good fit. gfi = 0.960, agfi = 0.949, nfi = 0.957, ifi = 0.978, cfi = 0.978, and tli= 0.974, all goodness-of-fit indicators, met the general criteria, indicating that the model developed in this study was valid and a good fit to the recovered data.

In addition, the KMO test values for the four latent variables were 0.824, 0.858, 0.884, and 0.862, which were all greater than the general criterion of 0.70, and the Bartlett’s sphericity test results also showed that the significance probability values were all 0.000 (*p* < 0.01), so the null hypothesis of Bartlett’s sphericity test could also be rejected and the validity structure could be considered as being good.

### 3.2. Structural Equation Model Results

In this study, structural equation model path analysis was performed using AMOS 21.0 software, which led to the structural equation model path coefficient values and C.R. values (Table 6).

#### 3.2.1. The Influence of Environmental Awareness on the Dimensions of Social Capital

The standardized path coefficient value for the influence of environmental awareness on social network was 0.003 (t-value = 0.035, *p* = 0.972 > 0.05), indicating that environmental awareness did not play a significant role in influencing social network (Table 6). These results did not support H.1a.

The standardized path coefficient value for the influence of environmental awareness on social trust was 0.198 (t-value = 2.905, *p* = 0.004 < 0.01), indicating a significant positive effect of environmental awareness on social trust. These results supported H.1b. The standardized path coefficient value for the influence of environmental awareness on social norms was 0.419 (t-value = 5.62, *p* = 0.000 < 0.001), indicating a significant positive effect of environmental awareness on social norms. These results supported H.1c. The standardized path coefficient value for the influence of environmental awareness on community belonging was 0.208 (t-value = 3.156, *p* = 0.002 < 0.01), indicating a significant positive effect of environmental awareness on community belonging. These results supported H.1d. The standardized path coefficient value for the influence of environmental awareness on community voluntarism was 0.298 (t-value = 4.258, *p* = 0.000 < 0.001), indicating that environmental awareness has a significant positive effect on community voluntarism. These results supported H.1e (Table 6).

#### 3.2.2. The Impact of Habitat Quality on the Dimensions of Social Capital

The standardized path coefficient value for the influence of habitat quality on social networks was 0.503 (t-value = 6.589, *p* = 0.000 < 0.001), indicating a significant positive effect of habitat quality on social networks. These results did not support H.2a. The standardized path coefficient value for the influence of habitat quality on social trust was 0.496 (t-value = 7.2, *p* = 0.000 < 0.001), indicating a significant positive effect of habitat quality on social trust. These results supported H.2b.The standardized path coefficient value for the influence of habitat quality on social norms was 0.284 (t-value = 4.136, *p* = 0.000 < 0.001), indicating that habitat quality has a significant positive effect on social norms. These results supported H.2c.The standardized path coefficient value for the influence of habitat quality sense of community belonging was 0.561 (t-value = 8.198, *p* = 0.000 < 0.001), indicating a significant positive effect of habitat quality on community belonging. These results supported H.2d. The standardized path coefficient value for the influence of habitat quality community voluntarism was 0.447 (t-value = 6.488, *p* = 0.000 < 0.001), indicating a significant positive effect of habitat quality on community voluntarism. These results supported H.2e (Table 6).

#### 3.2.3. The Influence of Environmental Awareness on Pro-Environmental Behavior

The standardized path coefficient value for the influence of environmental awareness on pro-environmental behavior was 0.246 (t-value = 2.785, *p* = 0.005 < 0.01), which can prove that environmental awareness has a significant positive effect on pro-environmental behavior, i.e., environmental awareness can significantly and positively predict pro-environmental behavior. These results supported H.3 (Table 6).

#### 3.2.4. The Influence of Habitat Quality on Pro-Environmental Behavior

The standardized path coefficient value for the influence of habitat quality on pro-environmental behavior was 0.285 (t-value = 2.615, *p* = 0.009 < 0.01). That indicates a significant positive effect of habitat quality on pro-environmental behavior. These results supported H.4 (Table 6).

#### 3.2.5. The Influence of Social Capital on Pro-Environmental Behavior

The standardized path coefficient value for the influence of social network on pro-environmental behavior was 0.019 (t-value = 0.464, *p* = 0.643 > 0.05), indicating that the effect of social network on pro-environmental behavior did not pass the significance test, namely, the effect of social networks on pro-environmental behaviors. These results did not support H.5a (Table 6).

The standardized path coefficient value for the influence of social trust on pro-environmental behavior was 0.134 (t-value = 2.69, *p* = 0.007 < 0.01), indicating a significant positive effect of social trust on pro-environmental behavior. The standardized path coefficient value for the influence of social norms on pro-environmental behavior was 0.102 (t-value = 2.01, *p* = 0.044 < 0.05), indicating a significant positive effect of social norms on significant positive effect. The standardized path coefficient value for the influence of community affiliation on pro-environmental behavior was 0.156 (t-value = 2.685, *p* = 0.007 < 0.01), this shows that community affiliation has a significant and positive impact on environmental behavior. These results supported H.5d. The standardized path coefficient value for the influence of community voluntarism on pro-environmental behavior was 0.19 (t-value = 3.376, *p* = 0.000 < 0.001), indicating that community voluntarism has a significant positive effect on pro-environmental behavior. These results supported H.5e (Table 6).

#### 3.2.6. The Mediating Effect of Social Capital

In order to verify whether there was a mediating effect of social capital in the influence path of environmental awareness and habitat environmental quality on pro-environmental behavior, the Bootstrap method in AMOS 21.0 software (IBM, New York, USA) was used to test the mediating effect, and then the mediating effect of each dimension of social capital in the influence mechanism of environmental awareness and habitat environmental quality on pro-environmental behavior was explored. The main analysis results are shown in Table 7.

Since neither environmental awareness on social networks nor social networks on pro-environmental behaviors played a significant role, the basic prerequisites of the mediating effect test were not satisfied, so no further mediating effect test was needed. However, in this study, the mediating effect was also tested in order to truly show the results. The results showed that the *p*-value of the mediating effect was greater than the standard 0.05, indicating that the mediating effect was not valid.

(1) Social capital has a mediating effect between the influence of environmental awareness on pro-environmental behavior.

Firstly, the [environmental awareness—social trust—pro-environmental behavior] effect value of 0.026, with 95% confidence upper and lower intervals of [0.002–0.072], does not contain 0, and the *p*-value is less than the significance level of 0.05, indicating the presence of a mediating effect. These results supported H.6b. Secondly, the [environmental awareness—social norms—pro-environmental behavior] effect value of 0.043, with a 95% confidence interval above and below [0.001–0.102] does not contain 0, and the *p*-value is less than the significance level of 0.05, indicating the existence of a mediating effect. These results supported H.6c. Thirdly, the [environmental awareness—community belongingness—pro-environmental behavior] effect value of 0.032, with 95% confidence upper and lower intervals of [0.005–0.076] does not contain 0, and the *p*-value is less than the significance level of 0.05, indicating the existence of a mediating effect. These results supported H.6d.Fourthly, the effect value for [environmental awareness—community voluntarism—pro-environmental behavior] was 0.057, with an upper and lower 95% confidence interval of [0.015–0.124] not containing 0, and a *p*-value of less than the significance level of 0.05, indicating the presence of a mediating effect. These results supported H.6e.

(2) Social capital has a mediating effect between the effects of habitat quality on pro-environmental behavior.

Firstly, the [habitat quality—social trust—pro-environmental behavior] effect value of 0.066, with 95% confidence upper and lower intervals of [0.014–0.138], does not contain 0, and the *p*-value is less than the significance level of 0.05, indicating the presence of a mediating effect. These results supported H.7b. Secondly, the [habitat quality—social norms—pro-environmental behavior] effect value of 0.029, with a 95% confidence interval above and below [0.001–0.075], does not contain 0, and the *p*-value is less than the significance level of 0.05, indicating the existence of a mediating effect. These results supported H.7c. Thirdly, the [habitat quality—community belongingness—pro-environmental behavior] effect value of 0.087, with a 95% confidence interval above and below [0.012–0.169], does not contain 0, and the *p*-value is less than the significant level of 0.05, indicating the existence of a mediating effect. These results supported H.7d. Fourthly, the effect value of [habitat quality—community voluntarism—pro-environmental behavior] is 0.085, the upper and lower 95% confidence interval of [0.031–0.172] does not contain 0, and the *p*-value is less than the significant level 0.05, indicating the existence of a mediating effect. These results supported H.7e. Habitat quality firstly acts directly on pro-environmental behavior, and secondly, it indirectly influences pro-environmental behavior with social trust, social norms, sense of community belonging, and community voluntarism. That is, social trust, social norms, community belongingness, and community voluntarism partially mediate the effect between habitat quality and pro-environmental behavior.

## 4. Discussion

This study focuses on the pro-environmental behavior of community residents, and constructs an analytical framework of “environmental awareness, habitat environment-social capital-pro-environmental behavior” from multiple perspectives, including external social environmental factors, situational factors, and internal social psychological factors. Using structural equation modeling, we analyzed the influence of environmental awareness and habitat on pro-environmental behavior. Based on this, we further verified the mediating effect of social capital dimensions in the influence mechanism of environmental awareness and habitat environment on pro-environmental behavior using the Bootstrap method. The main findings are as follows.

Regarding H1, environmental awareness has a significant effect on social trust, social norms, community belongingness, and community voluntarism in social capital. Among them, environmental awareness had the most significant effect on social norms, followed by community voluntarism, community belongingness, and social trust. Environmental awareness has no significant impact on social networks, which are not consistent with the results of Steg, L. et al., (2009) [46]. This may be due to the fact of that social networks are relatively stable social relationships formed by the interaction between residents, and in the complex social environment of “differential pattern” and vernacular relationship, the social network formed by the residents’ social interaction activities with their neighbors, relatives, and friends in their daily lives has a broad meaning. This network is usually fixed and will not change with changes in environmental awareness.

Regarding H2, the quality of the habitat environment has a direct effect on social capital and the most significant effect on the sense of community belonging, followed by social networks, social trust, and community voluntarism. However, the quality of the habitat environment has the least effect on social norms. This corroborates John A. K.’s view that the physical urban environment affects social capital formation [47]. The importance of habitat has also been confirmed by traditional Chinese cultural concepts. For example, in the Chinese idiom of “Anju Leye”, “Anju” is the basic condition for people to live and produce, and it is placed written first, indicating its importance: “Only when people live in peace can they live in happiness”.

Regarding H3, environmental awareness can significantly and positively predict pro-environmental behavior. This is consistent with the view put forward by Kang, M. et al., (2009) [48], which indicates that environmental awareness is one of the important driving factors affecting environment-friendly behavior. The mechanism of the effect may be as follows: first, the more community residents are inclined toward naturalistic concepts, the more likely they are to adopt daily environmental protection behaviors; second, community residents with more environmental knowledge will be more likely to understand the relationship between daily behaviors and environmental protection, and will also acquire more knowledge related to pro-environmental behaviors, thus driving people to adopt pro-environmental behaviors in their daily lives; third, when community residents have strong environmental values, they usually feel a high level of emotional attachment to the environment, develop a sense of closeness to the natural environment, take the initiative to connect with the natural world around them, and believe that they are closely related to the natural world around them. When this sense of connection to the environment increases, residents often increase their level of pro-environmental behavior [49].

Regarding H4, there was a significant positive effect of habitat quality on pro-environmental behavior. This conclusion is consistent with the view of Han, H. et al., (2017) [50] that the living environment is an external stimulus. When residents perceive the high-quality living environment, they will naturally restrict their own behavior, generate the mentality of environmental protection and make pro environmental behavior. The reason for this is that at high levels of habitat quality, residents choose to conform to environmental characteristics and self-regulate their own environmental behaviors [30], thus adopting pro-environmental behaviors; while at low levels of habitat quality, residents do not perceive external pressures on their inappropriate environmental behaviors and view them as normal and acceptable, thus inhibiting pro-environmental behaviors. In addition, a low level of habitat quality makes residents feel that pro-environmental behavior is difficult and ineffective, and therefore they will give up their personal efforts and choose to conform to the external environment, thus triggering inappropriate environmental behavior. On the other hand, high levels of habitat quality improve residents’ assessment of the effectiveness of their behaviors, thus leading them to adopt environmental behaviors [51].

Regarding H5, it shows that social trust, social norms, community belongingness, and community voluntarism in social capital can significantly promote residents’ pro-environmental behavior. The research conclusion confirms the opinion of Polyzou, E. et al., (2011) [52]. That is to say, the formation of social capital can guide the collective action of community members, and on this basis become a factor guiding individual action in the community, and also promote the pro-environmental behavior of community residents. Among them, the relationship between the effect size of each dimension of social capital is as follows: community voluntarism > community belongingness > social trust > social norms. The reason may be that if the degree of social capital is higher, community residents will be more active in complying with social norms, take the initiative to discipline their individual behavior, promote collective cooperation, and will monitor specific actions. When residents’ cognitive and moral beliefs are consistent with group norms, they identify with the group, making pro-environmental behavior a collective action of individuals with a common perspective (i.e., opinion-based group). In this process, residents have a stronger sense of emotional attachment to their community and are able to participate in community volunteerism, and help other residents for free, or are willing to help other residents for free, showing more positive pro-environmental behavior. Thus, increased awareness of environmental protection can produce good pro-environmental behaviors.

Regarding H6, the research results show that when the residents of a community are more environmentally conscious this motivates them to interact effectively on environmental issues, thus forming a collective environmental consciousness. Based on rational human assumption, individuals are more likely to display “free-rider” behaviors when making decisions, and enter into a collective action dilemma. This research result confirms the view of Putnam, R. D. et al., (1997) [53] that social capital is an important factor to encourage individuals to actively participate in cooperation and avoid the dilemma of collective action. Social capital is considered to be an important factor to motivate individuals to actively participate in cooperation and to avoid collective action dilemmas. When an awareness of group behavior is established, and under the influence of the mediating effect of social capital, it will have a positive impact on the pro-environmental behaviors of community residents. Therefore, while environmental awareness directly influences residents’ pro-environmental behaviors, it also indirectly influences their pro-environmental behaviors through the role of residents’ social capital. Therefore, the influence of environmental consciousness on pro-environmental behavior can be conveyed through environmental consciousness, and the logical relationship between the three is “environmental consciousness → social capital (social trust, social norms, community belonging, and community voluntarism) → pro-environmental behavior”.

Regarding H7, the mechanism of action may be that environmental awareness influences changes in residents’ social capital, which in turn promotes residents’ pro-environmental behavior. This research result is consistent with Petzold, J. et al., (2015) [54] and Hua, Y. et al., (2020) [54,55]. That is, satisfaction with the quality of the human environment will motivate community residents to interact effectively on environmental issues and be more willing to interact and communicate with each other. Based on the social exchange theory of reciprocity, in a peaceful community environment, residents will actively work for the well-being of their community, and the resulting emotional ties will stimulate the residents’ sense of community, which will increase their own social capital stock. In other words, social capital, as a collection of resources based on neighborhood interaction, trust, common ideas, and relationship networks, can promote residents’ spontaneous adoption of environmental sustainability protection behaviors and promote the construction of an environmental governance system with the joint participation of social organizations and residents.

In terms of samples, this study selected community residents in Jinan, Shandong Province. Jinan is one of the 15 sub-provincial cities in China, with a permanent population of 9.2024 million people. Therefore, the research conclusions drawn from Jinan City as a case area have certain reference value for exploring the problem of the pro-environment behavior of residents in large and medium-sized cities across the country. A more comprehensive studies in more places would further verify the generalization of the results found in this study. Therefore, more in-depth research needs to explore how to further refine the research framework and increase the number of completed spatial samples and questionnaires. Subsequently, in a future study, we will consider dividing pro-environmental behavior into multiple dimensions to explore the underlying mechanisms influencing the pro-environmental behavior of community residents in greater depth.

## 5. Conclusions

This study empirically analyzes the mechanism of environmental awareness and habitat quality on pro-environmental behavior, and identifies the important mediating effect of social capital. In other words, the social capital of community residents is influenced by the perception of individual environmental awareness and habitat quality; after taking on this influence, individuals will continue to act on their pro-environmental behavior. This study confirmed the multiple effects of environmental awareness as an internal personal psychological factor, habitat quality as an external contextual factor, and social capital as a social environmental factor on pro-environmental behavior.

For community residents, residents’ awareness of environmental issues affects their trust in society, their level of participation, etc., and makes it easy to build a sense of community with a particular environment in real life. Habitat is the spatial carrier of social capital formation, and with the satisfaction that community residents have with the habitat, they will implicitly internalize their sense of belonging to the community. As residents’ sense of belonging to the community and sense of community grows, social capital can be formed in spaces where community members can interact, and social capital is a “soft environment” that can improve the efficiency of social functioning and stimulate social vitality. This leads to civic behaviors that contribute to community development, which in turn promotes pro-environmental behaviors among community residents.

Based on this, the implementation of pro-environmental behaviors by community residents can be promoted from the following three aspects. One is to raise the environmental awareness of residents. Public education can be conducted in public places and government agencies on the current state of environmental protection, knowledge of protection, and significance and future development prospects, so that residents can understand the local environment, raise expectations for the achievement of group goals, and portray a better vision of collective efforts. The content of education should be relevant, practical, and actionable, so that residents can understand what they can do to have a beneficial impact on the environment. It is also important to expand residents’ knowledge of the environment, especially the knowledge and skills that can guide them in their daily environmental behavior. The second is to improve the quality of the habitat. The higher the quality of residents’ habitat environment, the greater the positive impact on pro-environmental behavior. Nowadays, local governments pay more attention to community hardware facilities, but relatively less investment in software facilities. Therefore, on top of focusing on hard environment construction, it is more important to pay attention to community human environment construction, to create a good cultural atmosphere for residents, and to create external conditions that are conducive to the implementation of pro-environmental behaviors. If these scenarios are described more specifically, contextual factors such as setting up convenient waste recycling bins for residents to separate garbage, and establishing an effective management system for residents to separate domestic waste are essential. These contextual factors will be an effective support for community residents to implement pro-environmental behaviors. The third is to cultivate the social capital of the population. To foster the concepts of altruism and social norms, we can actively carry out various popular education activities. To strengthen residents’ sense of belonging and identity to the area, communities can be guided to normalize various cultural and sports activities that stimulate the inner emotions. To enhance residents’ sense of dependence on the community, better community environmental and health conditions can be created, as well as better infrastructure conditions and community management conditions, so that the community can best meet the real needs of residents and become a warm community. In order to strengthen residents’ emotions towards the community, diversified community activities should be held and the public spaces for activities should be built. In addition, a platform for interaction to strengthen residents’ ties, reinforce collective memory, and eliminate barriers and strangeness should be provided.

## Figures and Tables

**Table 1:** Environmental awareness evaluation indicators.

Dimensionality	Indicators
Environmental Concern	You are concerned about the air problem in your place of residence. You are concerned about the water quality in your place of residence. You are concerned about the environmental problems of the soil in your place of residence (pollution caused by domestic garbage and waste). You are concerned about the noise problem in your place of residence. You are concerned about the greening of residential areas.
Environmental Knowledge	6. You understand many points related to environmental protection (such as that formaldehyde poses a threat to human health, laundry detergent containing phosphorus will cause water pollution, the use of excessive chemical fertilizers will damage the environment, etc.). 7. You consider the relationship between the environment and the development of economics and society. 8. You understanding the relationship between environment and health.
Environmental Values	9. You think everyone should participate in protecting the environment. 10. You become angry when seeing reports about environmental damage. 11. If the environment is not protected, it will lead to an environmental disaster. 12. You feel very uneasy when you think of the high probability that our next generation will live in a poor living environment.

**Table 2:** Habitat quality evaluation indicators.

Design Level	Indicators
Environmental health condition	Outdoor air quality satisfaction. Satisfaction with the degree of greening of the place of residence. Satisfaction with water quality and hygiene of living environment. Satisfaction with the noise pollution situation.
Infrastructure conditions	5. The construction status of cultural and recreational facilities. 6. The construction status of health service facilities (community health stations, outpatient clinics, pharmacies, etc.). 7. The extent of construction of fitness and leisure services facilities (activity centers, community libraries, etc.). 8. Construction status of daily living service facilities (parking lots, roads, sidewalks, delivery cabinets, direct drinking water facilities, etc.).
Environmental Management Status	9. The degree of personal and property security of the quality of the habitat. 10. Construction status of security service settings (monitoring, access control facilities, etc.). 11. The neatness of the living and building environment. 12. The neatness of the roads around the residential area.

**Table 3:** Social capital evaluation indicators.

Design Level	Indicators
Social Networks	Community neighbors will greet you when they meet you. You have many residents in your community who are well connected enough to visit you on a regular basis. You are regularly involved in various organizations in the community.
Social Trust	4. You trust the community council. 5. You can borrow things from your neighbor’s house very smoothly. 6. You believe that other residents will not pursue their own interests to your detriment.
Social Norms	7. Environmental protection system in the community is well established. 8. Community civilization norms are consciously abiding by community residents. 9. Those who violate the rules of community civility will be effectively punished.
Sense of Community Belonging	10. You are proud to be a resident of your community. 11. You feel at home in the community. 12. Do you want to continue living in this community?
Community volunteerism	13. You will actively contribute to the community and participate in the construction of the community. 14. You will actively participate in community volunteer services. 15. If a problem affects the harmony of the whole community, you will actively solve the problem together with other residents.

**Table 4:** Pro-environmental behavior evaluation indicators.

Dimensionality	Indicators
Ecological Management	You take care of the environment in public places (no littering, etc.). You will minimize the use of disposable personal items (disposable plastic bags, cutlery, etc.). You separate garbage that can be recycled (such as recycling plastic bottles, printing paper). You save energy (such as turning off lights with your hands, green travel). You save water. (e.g., turn off the water, use the water for washing dishes, water the flowers).
Consumer Behavior	6. You do not buy products that easily cause harm to the environment. 7. You buy energy-saving household appliances. 8. You buy goods with the green product mark, even if the price is more expensive than other similar goods.
Persuasive behavior	9. You encourage others to adopt pro-environmental behaviors, such as saving energy and water. 10. You advise others to stop damaging the environment, such as littering, sewage discharge. 11. You take the initiative to explain the importance of environmental protection to others. 12. You publicly express statements or opinions in support of environmental protection.
Civic Behavior	13. You participate in community discussions related to environmental issues. 14. You report to the relevant departments when you see acts that damage the environment. 15. You advise others not to violate laws and regulations related to environmental protection. 16. You proactively discuss with others on how to solve environmental problems.

**Table 5:** Descriptive statistics of the sample.

Basic Information	Classification	Frequency	Percentage
Gender	Male	543	54
	Female	462	46
Age	16–20 years old	91	9.1
	21–30 years old	272	27.1
	31–40 years old	192	19.1
	41–50 years old	202	20.1
	51–60 years old	141	14
	61 years old and above	107	10.6
Current place of residence	Lixia District	262	26.0
	Licheng District	304	30.2
	Changqing District	212	21.1
	Zhangqiu District	268	26.7
Average monthly income	Under RMB 4500	111	11
	RMB 4500–5500	272	27.1
	RMB 5500–6500	322	32
	RMB 6500–7500	181	18
	RMB 7500 and above	119	11.8
Highest level of education	Junior high school and below	119	11.8
	High school, junior college, technical school	213	21.2
	University specialists	336	33.4
	Undergraduate	228	22.7
	Graduate student and above	109	10.8
Current Occupation	Students	101	10
	Government units, institutions	162	16.1
	State-owned enterprises	151	15
	Private enterprises, foreign-funded enterprises	262	26.1
	Individual business people, personal studio	192	19.1
	Retirees and others	117	11.6

**Table 6:** Descriptive statistics of the sample.

Research Hypothesis Path			Standardization Factor	Standard Error	t	*p*
Environmental Awareness	→	Social Networks	0.003	0.155	0.035	0.972
Environmental Awareness	→	Social Trust	0.198	0.145	2.905	0.004
Environmental Awareness	→	Social Norms	0.419	0.143	5.62	***
Environmental Awareness	→	Sense of Community Belonging	0.208	0.139	3.156	0.002
Environmental Awareness	→	Community volunteerism	0.298	0.127	4.258	***
Habitat Quality	→	Social Networks	0.503	0.109	6.589	***
Habitat Quality	→	Social Trust	0.496	0.101	7.2	***
Habitat Quality	→	Social Norms	0.284	0.09	4.136	***
Habitat Quality	→	Sense of Community Belonging	0.561	0.1	8.198	***
Habitat Quality	→	Community volunteerism	0.447	0.086	6.488	***
Environmental Awareness	→	Pro-environmental behavior	0.246	0.072	2.785	0.005
Habitat Quality	→	Pro-environmental behavior	0.285	0.061	2.615	0.009
Social Networks	→	Pro-environmental behavior	0.019	0.016	0.464	0.643
Social Trust	→	Pro-environmental behavior	0.134	0.019	2.69	0.007
Social Norms	→	Pro-environmental behavior	0.102	0.022	2.01	0.044
Sense of Community Belonging	→	Pro-environmental behavior	0.156	0.022	2.685	0.007
Community volunteerism	→	Pro-environmental behavior	0.19	0.025	3.376	***

Note: *** indicates *p* < 0.001; “→” indicates the influence path.

**Table 7:** Bootstrap method-mediated effect test results.

Intermediary Effect Hypothesis	Effect Value	Standard Error	Lower 95% Confidence Interval	Upper 95% Confidence Interval	*p*
Environmental Awareness—Social Network—Pro-Environmental Behavior	0.001	0.006	−0.01	0.012	0.86
Environmental awareness—Social trust—Pro-environmental behavior	0.026	0.017	0.002	0.072	0.031
Environmental Awareness—Social Norms—Pro-Environmental Behavior	0.043	0.025	0.001	0.102	0.048
Environmental awareness—sense of community belonging—pro-environmental behavior	0.032	0.017	0.005	0.076	0.012
Environmental Awareness—Community Voluntarism—Pro-Environmental Behavior	0.057	0.027	0.015	0.124	0.004
Habitat Quality—Social Network—Pro-Environmental Behavior	0.01	0.024	−0.036	0.063	0.677
Habitat Quality—Social Trust—Pro-Environmental Behavior	0.066	0.031	0.014	0.138	0.016
Habitat Quality—Social Norms—Pro-Environmental Behavior	0.029	0.018	0.001	0.075	0.044
Habitat Quality—Community Belonging—Pro-Environmental Behavior	0.087	0.038	0.012	0.169	0.017
Habitat Quality—Community Voluntarism—Pro-Environmental Behavior	0.085	0.034	0.031	0.172	0.001

## Data Availability

Not applicable.
